# Economic Inequality Over the Life Course: Causes and Effects of Cumulative Dis/Advantage Processes

**DOI:** 10.1093/geroni/igaa057.1957

**Published:** 2020-12-16

**Authors:** Jessica Kelley, Stephen Crystal, Jessica Kelley

## Abstract

Economic inequality has grown rapidly in all age groups in the past several decades. In each successive cohort, the wealth gap grows for young people and seems to accelerate faster over the life course. While rising inequality has taken its toll on Baby Boomers, we have become acutely aware of the increasing economic pressures across the entire life course (work precarity; student loans) that will manifest in the greatest degree of inequality in older adulthood seen to date. This session explores the forces that have shaped the degree of inequality among current older adults and are setting the stage for future cohorts of older adults. Presenters will explore several aspects of this issue: the growing state of the “risk retirement,” the impact of income inequality on later-life wealth and health, the structural racism written into economic policies intended to help Americans accumulate wealth and maintain health, and the market disadvantage for GED recipients compared to high school diploma recipients.

